# Makulopathie bei Sichelzellerkrankung

**DOI:** 10.1007/s00347-020-01319-8

**Published:** 2021-01-27

**Authors:** Isabel Bachmeier, Christiane Blecha, Jürgen Föll, Daniel Wolff, Herbert Jägle

**Affiliations:** 1 Klinik und Poliklinik für Augenheilkunde, Universitätsklinikum Regensburg, Franz-Josef-Strauß-Allee 11, 93053 Regensburg, Deutschland; 2 Abteilung für Pädiatrische Hämatologie, Onkologie und Stammzelltransplantation, Universitätsklinikum Regensburg, Regensburg, Deutschland; 3 Klinik und Poliklinik für Innere Medizin III, Universitätsklinikum Regensburg, Regensburg, Deutschland

**Keywords:** Sichelzellmakulopathie, Makulaverdünnung, Retinopathie, Optikopathie, OCT-Angiographie, Sickle cell maculopathy, Macular thinning, Retinopathy, Optic neuropathy, OCT angiography

## Abstract

**Hintergrund:**

Die Sichelzellerkrankung (SZE) ist eine hereditäre Hämoglobinopathie, die durch rezidivierende vasookklusive Episoden zur Mikrozirkulationsstörung verschiedener Organsysteme mit teils letalem Ausgang führt. Bei der okulären Manifestation der SZE ist am bekanntesten die periphere Sichelzellretinopathie (SZR). Unabhängig davon kann es bereits früh im Krankheitsverlauf zur Sichelzellmakulopathie (SZM) kommen.

**Methoden:**

Review der internationalen und deutschsprachigen Literatur zur okulären Beteiligung bei SZE mit Fokus auf die SZR und SZM sowie Überblick über aktuelle systemische Therapieansätze bei SZE anlässlich der Vorstellung zweier Patienten mit HbSS-SZE.

**Ergebnis und Schlussfolgerung:**

Im Gegensatz zur SZR ist die SZM mit temporaler Verdünnung der inneren Netzhautschichten erst in den letzten 5 Jahren mit der Einführung von SD-OCT und OCTA vermehrt in die Literatur eingegangen. Unabhängig vom Vorliegen einer SZR kann es immerhin bei etwa der Hälfte der Patienten bereits früh im Krankheitsverlauf zu einer SZM kommen. Das Krankheitsbild wird auch in Deutschland durch den Fortschritt der systemischen Therapiemöglichkeiten und aufgrund von Migration präsenter werden. Durch Wissen um diese Komplikation der SZE kann eine frühzeitige Diagnosestellung erfolgen und unnötige Diagnostik vermieden werden.

## Abstract (en)

**Background:**

Sickle cell disease (SCD) is a hereditary hemoglobinopathy, which leads to microcirculatory disturbances of various organ systems through recurrent vaso-occlusive episodes, with a possibly fatal outcome. Sickle cell retinopathy (SCR) is the best described ocular manifestation of SCD. Irrespective of the presence of peripheral SCR, sickle cell maculopathy (SCM) can occur early in the course of the disease.

**Methods:**

Review of the international and German literature on ocular involvement in SCD with a focus on SCR and SCM and an overview of current systemic therapeutic approaches in SCD on the occasion of the presentation of two patients with HbSS SCD.

**Results and conclusion:**

In contrast to SCR, SCM with temporal thinning of the inner retinal layers has only been increasingly described in the literature in the last 5 years, with the advent of SD-OCT and OCTA. Irrespective of the presence of SCR, as many as about half of the patients may develop SCM early in the course of the disease. As a result of progress in systemic therapeutic options and due to migration, the clinical picture will occur more often also in Germany. By knowing about this complication of SCD an early diagnosis can be made and unnecessary diagnostics can be avoided.

Der nachfolgende Artikel wird Ihnen verraten, warum eine Erkrankung, die vorwiegend im „fernen Afrika“ anzutreffen ist und die roten Blutkörperchen betrifft, auch einem deutschen Augenarzt begegnen kann und deshalb auch Ihr Interesse wecken dürfte.

## Hintergrund

### Epidemiologie, Pathogenese und klinische Manifestation der Sichelzellerkrankung

Die Sichelzellerkrankung (SZE) zählt zu den weltweit häufigsten und klinisch bedeutsamsten erblichen Hämoglobinopathien, für welche ca. 5 % der Weltbevölkerung Mutationsträger sind [48, 59]. Pro Jahr sind ca. 300.000 Neugeborene, davon 75 % in subsaharischen Regionen Afrikas, betroffen. Weitere Endemiegebiete sind Indien, der Nahe Osten und der (vorwiegend östliche) Mittelmeerraum (u. a. Süditalien, Balkan, Griechenland, Türkei). Durch Migration aus endemischen Gebieten findet man auch in nördlicheren europäischen Ländern eine steigende Prävalenz der Erkrankung [59]. In Deutschland wird die aktuelle Prävalenz der SZE auf ca. 3000 geschätzt mit einer steigenden Tendenz durch Migranten aus Syrien, dem Irak und Zentralafrika [37, 38].

Bei der SZE handelt es sich um eine monogenetisch vererbte Hämoglobinopathie durch eine Punktmutation im β‑Globin-Gen auf Chromosom 11p15.5. Der Austausch einer einzelnen Aminosäure (β6 Glu → Val) des normalen Hämoglobin HbA verändert die Eigenschaften des so entstandenen anormalen Hämoglobin HbS derart, dass es bei Desoxygenierung polymerisiert [19]. Dies führt zur namensgebenden sichelförmigen Veränderung der Erythrozyten. Die sog. Sichelzellen sind fragil und weniger verformbar, was zu Hämolyse und rezidivierenden schmerzhaften vasookklusiven Krisen führt. Darüber hinaus werden durch Induktion von oxidativem Stress inflammatorische Prozesse in Gang gesetzt, woraus eine chronische Vaskulopathie resultiert. Diese kann die Mikrozirkulation aller Organsysteme betreffen und u. a. zu Niereninsuffizienz, Leberzirrhose, pulmonaler Hypertonie, ischämischen und hämorrhagischen ZNS-Infarkten oder Osteonekrosen führen [59]. Betroffene Patienten versterben häufig an kardiovaskulären, respiratorischen oder zerebrovaskulären Komplikationen. Der Genotyp beeinflusst den Phänotyp, wobei der homozygote HbSS-Genotyp und der gemischt heterozygote HbSβ^0^ Thal(β-Thalassämie)-Genotyp tendenziell mit schwereren klinischen Verläufen assoziiert sind als der gemischt heterozygote HbSC-Genotyp (HbC entsteht durch die Substitution β6 Glu → Lys) [76].

Aufgrund des komplexen und weitgreifenden klinischen Bildes hat sich die Bezeichnung „Sichelzellerkrankung“ durchgesetzt. Nur der HbSS-Genotyp wird im englischen Sprachraum noch als „Sichelzellanämie“ bezeichnet.

### Okuläre Manifestation

Die okuläre Manifestation umfasst u. a. kommaförmige Bindehautgefäße, ein Sekundärglaukom durch Hyphäma oder Rubeosis iridis und orbitale Komplikationen [18, 57]. Weitaus bekannter sind die retinalen Komplikationen, wobei man eine proliferative und eine nichtproliferative Sichelzellretinopathie (SZR) unterscheidet [77]. Unabhängig davon kann eine Makulopathie auftreten [46]. Zur Veranschaulichung dienen 2 Fallvorstellungen von Patienten mit SZE, bevor auf die verschiedenen Formen der retinalen Beteiligung bei SZE detailliert eingegangen wird.

## Fallvorstellung 1

**Figure 1:**
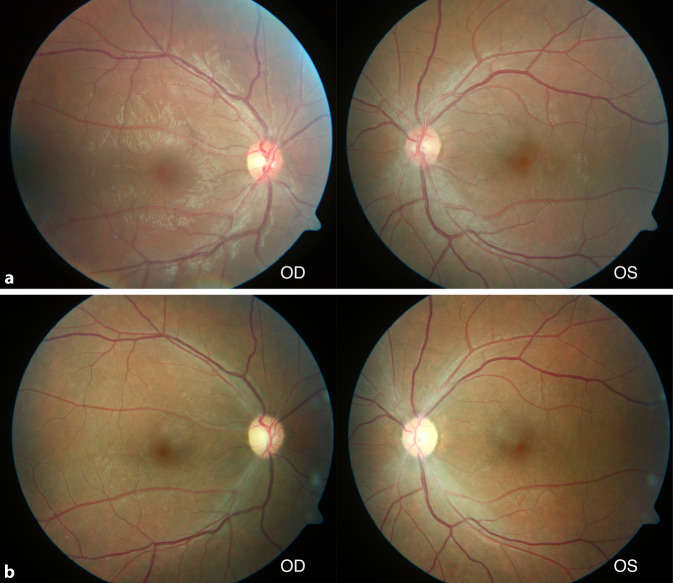

**Figure 2:**
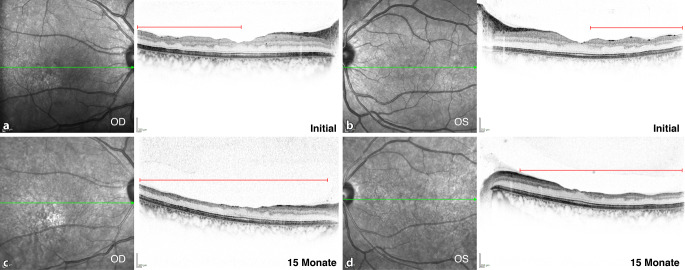

**Figure 3:**
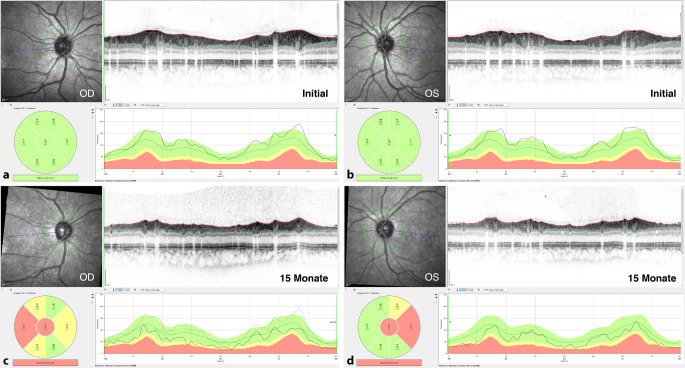

In der Augenklinik des Universitätsklinikum Regensburg wurde eine 20-jährige Patientin mit seit der Kindheit bekannter HbSS-SZE vor geplanter Stammzelltransplantation (HSCT [„hematopoietic stem cell transplantation“]) untersucht. Die Indikation zur HSCT wurde wegen zahlreicher Komplikationen der SZE mit teils schwerwiegenden Komplikationen an verschiedenen Organsystemen (multiple Schmerzkrisen, akute Thoraxsyndrome, Lungenembolien, ZNS-Ischämien, multiple Osteonekrosen) gestellt. Die Patientin war okulär beschwerdefrei mit einem unkorrigierten Visus von 1,0 beidseits. Spaltlampenmikroskopisch zeigten sich unauffällige Befunde mit klaren brechenden Medien. Die Fundoskopie ergab vitale Sehnerven mit unauffälliger zentraler und peripherer Netzhaut (Abb. 1a) ohne Hinweise auf eine Sichelzellretinopathie oder angioide Streifen. Überraschenderweise zeigte sich in der OCT eine Verdünnung der zentralen Netzhaut, die insbesondere die inneren Netzhautschichten betraf und im temporalen Makulabereich besonders ausgeprägt war (Abb. 2a). Aufgrund der allgemeinen Situation der Patientin musste auf Spezialuntersuchungen verzichtet werden. Die Patientin wies im weiteren Verlauf eine milde okuläre Graft-versus-Host-Disease (GvHD) auf, welche mit Tränenersatzmitteln und später auch Ciclosporin A AT (Ikervis®, Santen GmbH, München, Deutschland) behandelt wurde, und berichtete von einer intermittierenden Visusminderung. Es bestanden leicht schwankende Visuswerte rechts zwischen 0,6 und 1,0 und links zwischen 0,8 und 1,0, die 15 Monate später auf 0,5 abgefallen waren. Die Verdünnung der zentralen Netzhaut war fortgeschritten und betraf nun den gesamten Makulabereich (Abb. 2b). Es zeigte sich fundoskopisch beidseits auch eine Papillenabblassung (Abb. 1b) mit Abnahme der retinalen Nervenfaserschichtdicke (RNFL) im Papillen-OCT (Abb. 3). Im ergänzten Muster-ERG zeigten sich reduzierte Amplituden und im Muster-VEP keine reproduzierbare Wellenform. Eine Literaturrecherche erbrachte schließlich eine Übereinstimmung der vorliegenden Veränderungen mit Beschreibungen der Sichelzellmakulopathie (SZM).

## Fallvorstellung 2

**Figure 4:**
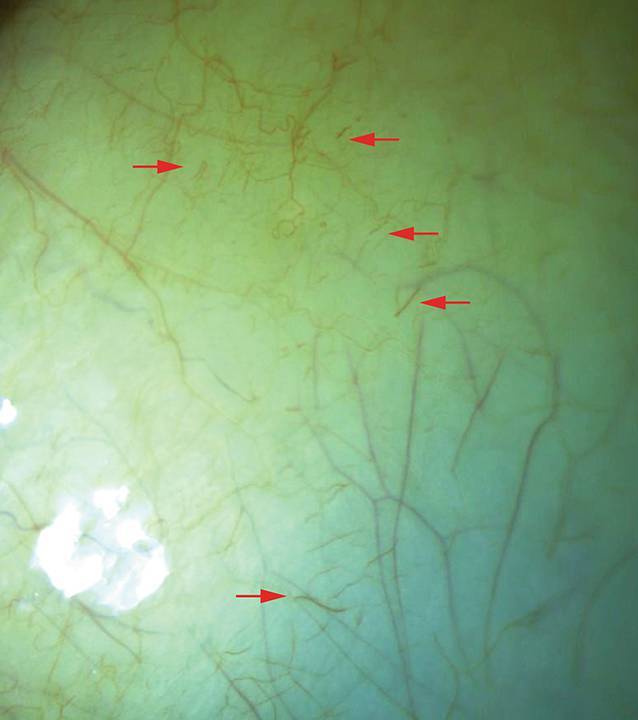

**Figure 5:**
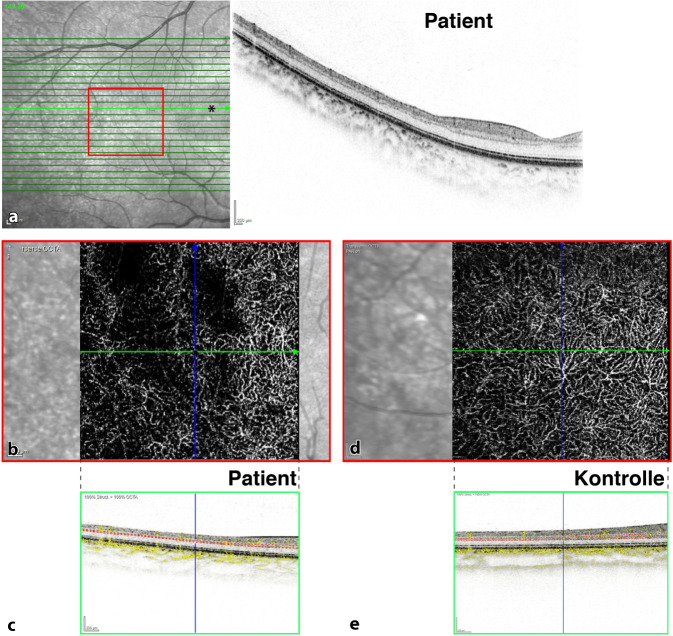

Ein junger Patient (12 Jahre) hatte eine seit 10 Jahren bekannte HbSS-SZE mit multiplen Komplikationen (u. a. Schmerzkrisen, eine aplastische Krise nach Parvo-B19-Infektion und erste Hinweise einer Sichelzellnephropathie mit Proteinurie), weshalb er bereits mehrfach Bluttransfusionen erhalten hatte. Er war ebenfalls ophthalmologisch asymptomatisch und wies einen unkorrigierten Visus von beidseits 1,0 auf. Spaltlampenmikroskopisch zeigten sich kommaförmige Bindehautgefäße (Abb. 4). Retinale Veränderungen (insbesondere Zeichen einer peripheren SZR oder angioide Streifen) bestanden nicht. Die OCT ergab insbesondere am rechten Auge eine temporale Makulaverdünnung, die in der OCT‑A mit nicht perfundierten Arealen im tiefen Kapillarplexus (DCP [„deep capillary plexus“]) korrespondierte (Abb. 5).

## Sichelzellretinopathie

Bei der SZR unterscheidet man proliferative und nichtproliferative Veränderungen [77]. Die von Goldberg 1971 etablierte Stadieneinteilung der proliferativen SZR (PSR) umfasst 5 Stadien (Tab. 1; [25]). Die Stadien I und II stellen streng genommen nichtproliferative Stadien dar und bezeichnen periphere nicht perfundierte Netzhautareale (Stadium I) und die Ausbildung arteriovenöser Gefäßanastomosen (Stadium II). Das Stadium III ist durch Tiefseefächer-artige Neovaskularisationen („sea fans“) an der Grenze von vaskularisierter zu nicht vaskularisierter Netzhaut gekennzeichnet. Diese okkludieren und bilden sich im Gegensatz zu Proliferationen anderer Genese (z. B. diabetische Retinopathie) in einem hohen Prozentsatz spontan zurück (Autoinfarzierung)[16]. Sie können jedoch auch zur Glaskörperblutung (Stadium IV) oder traktionsbedingten bzw. rhegmatogenen Netzhautablösung (Stadium V) führen. Angaben zur Prävalenz der PSR schwanken stark zwischen verschiedenen Studien und sind von verschiedenen Faktoren abhängig. Generell nimmt die Prävalenz mit dem Alter zu [7, 42]. Patienten mit dem HbSC-Genotyp erleiden im Vergleich zum HbSS-Genotyp häufiger eine PSR und schwerere Verläufe einer PSR [42]. Die Schwere der PSR korreliert auch mit der Schwere des systemischen klinischen Verlaufs [26, 42].

**Table 1:** 

Stadium I	Periphere arterioläre Okklusionen
Stadium II	Arteriovenöse Anastomosen
Stadium III	Neovaskularisationen („sea fans“)
Stadium IV	Glaskörperblutung
Stadium V	Netzhautablösung (traktiv/rhegmatogen)

Neben den genannten Befunden der PSR beinhaltet die retinale Beteiligung auch nichtproliferative Veränderungen wie sog. Lachsflecken („salmon patches“). Diese stellen meist unter der ILM gelegene durch Hämolyse lachsfarbene Blutungen dar, die spurlos abheilen können oder sog. Glitzerflecken („iridescent spots“) hinterlassen können. Es können aber auch durch reaktive fokale RPE-Hyperplasie und -Migration pigmentierte chorioretinale Narben, sog. schwarze Sonnenflecken („black sun bursts“), entstehen [21].

Es wurde auch ein gehäuftes Auftreten einer Tortuositas vasorum [70] und von „angioid streaks“ bei SZE beschrieben [51].

## Sichelzellmakulopathie

Bereits in den 70er-Jahren wurden makuläre Veränderungen im Rahmen einer SZE beschrieben. Romayanada et al. zeigten 1973 in histologischen Untersuchungen eine Verdünnung der inneren Netzhautschichten (Ganglionzell- und innere Körnerschicht) [63]. Im Jahr 1978 beschrieb Goldbaum mit dem Begriff „retinal depression sign“ eine fundoskopisch anomale Reflektivität der ILM infolge einer perfusionsbedingten Verdünnung der inneren Netzhaut [24]. Auch fluoreszeinangiographisch wurden bei SZE Gefäßveränderungen im Makulabereich, eine vergrößerte foveoläre avaskuläre Zone und nicht perfundierte perifoveale Areale dargestellt [3, 5, 70]. Schon in diesen Arbeiten zeigte sich eine Prädilektion der temporalen Makula. Eine Erklärung lieferten Stevens et al., die die Gefäße im Bereich der temporalen Raphe als Endgefäße beschrieben und dementsprechend die von ihnen perfundierten Netzhautareale als eine Art Wasserscheide der retinalen Perfusion [70].

Eine temporale Verdünnung der Makula in der SD-OCT wurde erstmals von Murthy et al. im Jahr 2011 bei asymptomatischen Patienten mit vollem Visus beschrieben [50]. Im selben Jahr veröffentlichten Hoang et al. eine gehäuft bei SZE-Patienten beobachtete zentrale Makulaverdünnung mit Aufweitung der fovealen Depression („foveal splaying“) [33]. Diese Veränderung wurde später von Mathew et al. jedoch eher als Normvariante bei Dunkelhäutigen und bei Patienten afrikanischer Abstammung gewertet [46].

Schließlich wurde, ebenfalls bei asymptomatischen Patienten, mittels OCT‑A nachgewiesen, dass die Atrophie auf eine Perfusionsstörung vornehmlich im DCP zurückzuführen ist [30, 31], also auf einer fokalen arteriolären Okklusion beruht. Es konnte gezeigt werden, dass auch asymptomatische Patienten in der (Mikro‑)Perimetrie zentrale und parazentrale Skotome [14] und Einschränkungen im Farb- und Kontrastsehen [44] aufweisen. Hierbei korrespondierten die Gesichtsfeldbereiche herabgesetzter und fehlender Sensitivität mit Nonperfusionsarealen im DCP [65]. Auch elektrophysiologisch ließen sich bei asymptomatischen Patienten Makulafunktionsstörungen mittels multifokalem ERG nachweisen [8].

Aus einer Studie von Martin et al. geht hervor, dass die SZM bereits früh in der Kindheit auftritt [45] und damit deutlich früher als die periphere SZR, für die der Gipfel des Auftretens im Jugend- oder frühen Erwachsenenalter liegt [7]. Die Prävalenz der SZM wird mit bis zu 64 % der SZE-Patienten angegeben [45]. Sie betrifft Patienten mit HbSS-SZE möglicherweise häufiger als Patienten mit HbSC-SZE [46], während es sich bei der PSR genau andersherum verhält [42]. Auch wenn eine temporale Makulaverdünnung unabhängig vom gleichzeitigen Vorliegen einer PSR auftreten kann, so scheint sie dennoch mit dem Grad peripherer Nonperfusion zu korrelieren und mit einem höheren Risiko einer PSR vergesellschaftet zu sein [46]. Auch wurde das Vorliegen einer SZM mit einem vermehrten Auftreten von zerebrovaskulären Komplikationen assoziiert, wodurch die SZM trotz asymptomatischen Verlaufs und trotz fehlender Therapieoptionen klinische Relevanz erlangt [45].

## Optikusneuropathie und parapapilläre Nervenfaserschicht

Zusätzlich zur Makulaverdünnung wies der von uns beschriebene Fall 1 im Verlauf eine Papillenabblassung mit Abnahme der parapapillären RNFL auf.

Vielfach wurde postuliert, dass Patienten mit SZE einem erhöhten Risiko unterliegen, ausgelöst durch einen Augeninnendruckanstieg eine Ischämie im Bereich des Sehnerven zu erleiden [27]. Zudem wurde ein erhöhtes Risiko für eine ischämische Optikopathie im Rahmen nichtokulärer Operationen beschrieben [64]. Es wurden auch Kasuistiken zu sich spontan entwickelnden AIONs [39] und PIONs [58, 69] veröffentlicht.

Darüber hinaus kann eine chronische Mikroangiopathie der den Sehnervkopf versorgenden Gefäße, vergleichbar mit der diabetischen Optikopathie, im Verlauf zur Papillenabblassung führen [68]. Bei SZE-Patienten ohne Glaukom wurde eine Abnahme der parapapillären RNFL mittels OCT beobachtet, deren Ausmaß mit der Schwere der Makulopathie [15] und deren Progredienz mit dem Vorhandensein zerebrovaskulärer Komplikationen [72] korrelierte. Auch für andere vaskuläre Retinopathien (Diabetes, arterieller Verschluss, HIV-Mikrovaskulopathie) wurde eine Abnahme der parapapillären RNFL beschrieben [4, 41, 56].

## Differenzialdiagnostische Überlegungen

### Medikamentennebenwirkungen

Häufig bei SZE verabreichte Medikamente sind Substanzen aus der Gruppe der Chelatbildner. Bei wiederholt notwendigen Bluttransfusionen überschreitet die anfallende Menge an Eisen die Kapazität des Körpers, Eisen zu binden und auszuscheiden. Der potenziell für sämtliche Körperzellen toxische erhöhte Spiegel ungebundenen Eisens kann durch Verabreichung chelatbildender Substanzen gebunden und über den Urin oder die Fäzes ausgeschieden werden. Verfügbare Eisenchelatoren sind Deferoxamin und Deferasirox [54].

In Zusammenhang mit dem seit fast 60 Jahren eingesetzten Deferoxamin wurden RPE-Veränderungen teils mit Akkumulation von Material im Bereich der äußeren Retina, eine Schießscheibenmakulopathie, eine Verdickung der Bruch-Membran und seltener eine pseudovitelliforme Makulopathie beschrieben [10].

Für das seit 2002 verfügbare Deferasirox wurden milde makuläre Pigmentveränderungen [75], eine perifoveale Verdünnung der äußeren Netzhaut mit Unterbrechung der ellipsoiden Zone ähnlich frühen OCT-Veränderungen unter Hydroxychloroquin-Einnahme [54] und eine Zunahme einer bereits vorbestehenden Deferoxamin-induzierten pseudovitelliformen Makulopathie nach Wechsel auf Deferasirox [10] beschrieben. Die Häufigkeit des Auftretens einer Makulopathie wird in der Fachinformation mit 1:1000 bis 1:100 angegeben.

Die Deferoxamin- und Deferasirox-assoziierten Veränderungen und Funktionsstörungen sind nach Absetzen und Dosisreduktion teilweise reversibel. Diskutiert wird, dass die Schädigung auf einem direkten toxischen Effekt auf das RPE beruht oder indirekt auf eine Chelatbildung mit für das RPE essenziellen Spurenelementen (Zink, Kupfer) zurückzuführen ist [10]. Während bei der Makulopathie durch Chelatbildner Veränderungen im Bereich der äußeren Netzhaut teils mit Materialablagerung im Vordergrund stehen, ist die SZM durch eine Verdünnung der inneren Netzhautschichten ohne Ablagerungen gekennzeichnet.

Abraham et al. postulierten zudem, dass auch eine Eisen- und Ferritinablagerung selbst durch einen toxischen Effekt zur irreversiblen Schießscheibenmakulopathie führen kann. Die Annahme stützt sich jedoch auf eine Einzelfallbeobachtung [2].

Auch andere bei SZE eingesetzte Medikamente können Makulopathien verursachen. Ding et al. fanden histologisch eine Atrophie der inneren Netzhaut mit einem massiven Untergang retinaler Ganglienzellen nach Einnahme des Purinanalogons Fludarabin, welches als Zytostatikum zur Vorbereitung vor HSCT eingesetzt wird [17]. Die Patienten hatten jedoch eine akute Visusminderung über wenige Wochen bis auf Lichtscheinwahrnehmung bzw. fehlende Lichtscheinwahrnehmung erlitten.

Für das Antimykotikum Fluconazol wurde die Entstehung eines zystoiden Makulaödems nach 1‑jähriger oraler Einnahme beschrieben [43]. Ein Makulaödem konnte bei der von uns in Fall 1 vorgestellten Patientin auch nach Einnahme zu keinem Zeitpunkt festgestellt werden.

### Retinale Komplikationen nach HSCT

Nach HSCT entwickeln bis zu 10 % der Patienten eine retinale Mikroangiopathie, die auch eine Netzhautischämie beinhalten kann [36]. Ursächlich ist eine Kombination aus Grunderkrankung, vorbereitender Therapie (Zytostatika, Bestrahlung) und GvHD. In der Regel zusätzlich vorliegende intraretinale Blutungen, Mikroaneurysmata, Cotton-wool-Herde oder harte Exsudate wurden bei der vorgestellten Patientin (Fall 1) zu keinem Zeitpunkt beobachtet. Die HSCT kann jedoch das rasche Fortschreiten der Makulaatrophie genauso wie die sich entwickelnde Papillenabblassung begünstigt haben.

Es werden im Zusammenhang mit HSCT selten Optikusneuropathien beschrieben wie auch bei der retinalen Mikroangiopathie auf dem Boden der Grunderkrankung, der vorbereitenden potenziell neurotoxischen Medikamente (wie das bereits erwähnte Zytostatikum Fludarabin) und der GvHD [36].

### Zustand nach manifestem Arterienastverschluss

Der Verschluss eines makulaversorgenden Arterienasts führt nach einem initialen Nervenfaserödem im Verlauf ebenfalls zur Atrophie und Verdünnung im Bereich der inneren Netzhautschichten [41]. In der Regel ist diese dann aber nicht asymptomatisch, sondern mit einem anamnestisch akuten Visusverlust und Zentralskotom vergesellschaftet [32].

### Parazentrale akute mittlere Makulopathie (PAMM)

Der erstmals 2013 beschriebenen PAMM liegt ein Verschluss im Bereich des DCP zugrunde, sie ist assoziiert mit retinalen Gefäßerkrankungen, und als Auslöser gelten u. a. systemische Hypovolämie, virale Infekte, Migräne und Trauma [61]. Während in der OCT initial eine fokale bandförmige Hyperreflektivität vorwiegend auf Höhe der inneren Körnerschicht besteht, entwickelt sich innerhalb von Monaten an dieser Stelle eine Verdünnung der inneren Körnerschicht und der äußeren plexiformen Schicht, begleitet von einer Verdickung der äußeren Körnerschicht. Insgesamt resultiert eine fokale Exkavation der inneren Netzhautoberfläche [52]. Im Allgemeinen ist sie im Akutstadium mit einer umschriebenen flauen gräulichen Fundusaufhellung verbunden und wird mit dem akuten Auftreten eines Skotoms symptomatisch. Aufgrund des identischen Pathomechanismus (Verschluss im DCP) und der späteren Entwicklung einer Atrophie im Bereich der inneren Netzhaut wird die PAMM von manchen Autoren sogar als die der SZM zugrunde liegende Vorläuferläsion diskutiert [34].

### Alport-Syndrom

Das Alport-Syndrom ist eine hereditäre Basalmembranerkrankung, der ein Strukturdefekt von Kollagen-IV-Fasern zugrunde liegt und die vorwiegend die renalen Glomeruli betrifft. Sie geht mit einem progressiven Nierenversagen einher, häufig besteht eine Assoziation mit einer progredienten Innenohrschwerhörigkeit [29]. Die okuläre Manifestation umfasst neben einem Lenticonus anterior, einer Katarakt, kornealen Trübungen und retinalen Flecken auch eine Makulopathie [67]. Es wurde eine Verdünnung der temporalen Makula beschrieben, wie bei der SZM überwiegend im Bereich der inneren Netzhautschichten, wobei diese aber gleichmäßiger verdünnt sind als bei der SZM [66, 74]. Eine unregelmäßige Verdünnung der inneren Netzhautschichten bei ebenfalls beschriebenen Makulaschichtforamina ähnelt im Aspekt eher der SZM, ist aber auf die Fovea beschränkt [67].

## Ophthalmologische Therapieoptionen

### Behandlung bei erhöhtem Augeninnendruck

Die Ausbildung eines Hyphämas oder einer Rubeosis iridis kann zu einem Sekundärglaukom und aufgrund der ohnehin erhöhten Anfälligkeit für Gefäßverschlüsse rasch zur ischämischen Optikusneuropathie oder zum Zentralarterienverschluss führen [27]. Ob bereits ein Augeninnendruck von 25 mmHg Anlass für chirurgische Interventionen (z. B. Parazentese und Vorderkammerspülung bei Hyphäma) geben sollte, ist jedoch umstritten [40]. Wichtig ist es, Carboanhydrasehemmer zu vermeiden, da die damit einhergehende systemische Dehydratation bzw. Azidose zu erhöhter Blutviskosität in der Mikrozirkulation führen bzw. die Sichelzellbildung verstärken kann [20]. Dass dies auch für topisch applizierte Carboanhydrasehemmer (Dorzolamid, Brinzolamid) gelten könnte, ist denkbar – die Augentropfen könnten lokal in der Vorderkammer bei Hyphäma eine Sichelzellbildung verursachen mit Behinderung des Durchtritts der Sichelzellen durch das Trabekelmaschenwerk –, konnte bisher jedoch nicht nachgewiesen werden [40].

### Laserbehandlung

Ziel einer Laserung ist es, eine Glaskörperblutung oder Traktionsablatio, also eine Progression der PSR zu Stadium IV bzw. V, zu verhindern. Frühere Ansätze, die „feeder vessels“ der Neovaskularisationsmembranen direkt zu koagulieren, wurden mittlerweile verlassen, da dies potenziell eine Glaskörperblutung, Netzhautrisse oder choroidale Neovaskularisationen verursachen kann. Vielmehr empfiehlt man bei „sea fans“ mit ausbleibender spontaner Autoinfarzierung eine zirkuläre disseminierte Photokoagulation oder eine gezielte Laserung im Bereich der ischämischen Netzhautareale [62].

### Anti-VEGF-Therapie

Auch eine intravitreale Anti-VEGF-Therapie kann möglicherweise die Rückbildung von Neovaskularisationen und Glaskörperblutungen bewirken, ggf. auch als präoperative Eingabe vor einer geplanten ppV [11, 47, 49]. Allerdings gibt es keine klinischen Studien, die eine Anti-VEGF-Therapie mit einer Scatter-laser-Therapie vergleichen. Als mögliche Komplikation einer Anti-VEGF-Therapie wurde die Entwicklung eines Hyphäma beschrieben [6].

### Pars-plana-Vitrektomie

Eine ppV ist bei nicht aufklarender Glaskörperblutung oder Ablatio indiziert. Es wurde hierbei eine erhöhte Rate von Ablatiorezidiven, iatrogenen Foramina und postoperativen Hyphämata oder Vorderabschnittischämien beschrieben [13]. Zur Dissektion der Neovaskularisationsmembranen wird eher zu Segmentations- als zu Delaminationstechniken geraten, da bei Letzteren aufgrund stark adhärenter Membranen ein erhöhtes Risiko für iatrogene Netzhautrisse besteht [78].

Die PSR wird im Allgemeinen nur selten therapiebedürftig, zugleich liegen keine klaren therapeutischen Leitlinien oder Empfehlungen aufgrund schwacher Evidenz vor. Einen umso höheren Stellenwert hat die Behandlung der Grunderkrankung. Diese kann auch einem Auftreten retinaler Komplikationen bzw. schwerer Verläufe zuvorkommen [1].

## Systemische Therapieoptionen

Das einzige krankheitsmodifizierende Medikament in Europa ist Hydroxycarbamid, das ab dem 2. Lebensjahr zugelassen ist (nach neuen Leitlinien empfohlen bereits ab 9. Lebensmonat). Unter anderem induziert es eine vermehrte Bildung von fetalem Hämoglobin (HbF), welches anders als das mutierte HbS nicht polymerisiert, sodass die Deformierung der Erythrozyten verhindert wird. Hierdurch wird die mediane Inzidenz schmerzhafter Krisen pro Jahr um 44 % reduziert [12, 60]. (Austausch‑)Transfusionen reduzieren die Konzentration an HbS und sind zur Behandlung bestimmter Akutkomplikationen erforderlich, können aber auch Teil eines langfristig ausgelegten Dauertherapiekonzepts v. a. bei Patienten mit ZNS-Komplikationen in Kombination mit Hydroxycarbamid sein [9]. In Erprobung sind mehrere neuere Medikamente, die die pathophysiologischen Mechanismen der Vasookklusion bei SZE lindern sollen, wie z. B. Voxelotor, Crizanlizumab und Rivipansel, die die Häufigkeit von Vasookklusion, Krankenhausaufenthalt und Schmerzmittelkonsum zu verringern scheinen [53, 73].

Zurzeit ist die allogene hämatopoetische Stammzelltransplantation (HSCT) für Patienten mit schwerwiegenden Komplikationen der SZE (Schlaganfälle, akute Thoraxsyndrome, signifikante andere Organschäden) die einzige angebotene kurative Therapieoption. Die myeloablative HSCT mit Knochenmark eines HLA-identischen Geschwisterspenders (MSD) ist derzeit Standard der Behandlung mit einem Gesamtüberleben von 90 %, ereignisfreiem Überleben von 80 % bei einer behandlungsbedingten Mortalität von 7 % [23, 55]. Jedoch liegt die allgemeine Verfügbarkeit von MSD bei unter 14 %, und die Wahrscheinlichkeit, einen passenden nichtverwandten Fremdspender (MUD) zu finden, liegt bei SZE-Patienten afrikanischer Herkunft unter 18 % [28, 71]. Teilweise HLA-nicht-übereinstimmende Spender ersten Grades (sog. haplo-HSCT) können den Spenderpool auf durchschnittlich 2,7 pro Patient erhöhen [22].

Eine weitere Therapieoption ist die Gentherapie, bei der eigene (autologe) hämatopoetische Stammzellen des Patienten gewonnen und im Labor genetisch modifizierte β-Ketten (HbA^T87Q^, „gene addition“) mithilfe eines lentiviralen Vektors dauerhaft im Genom integriert werden [35]. Die korrigierten Zellen werden dem Patienten dann nach einer myeloablativen Chemotherapie wieder zugeführt. Theoretisch ist ein kurativer Ansatz durch eine homologe Rekombination („gene correction“), also durch einen Austausch der Nukleotide, möglich. Dieser Goldstandard befindet sich noch in der vorklinischen Entwicklung. Eine weitere sehr interessante Methode ist die Induktion von HbF durch eine sog. „Genschere“. Dabei wird genomische DNA mit hoher Präzision modifiziert, um Insertionen sowie Deletionen (Indels) in die Erythroid-Enhancer-Region von BCL11A zu induzieren. Der Transkriptionsfaktor BCL11A, ein Regulator der HbF-Expression, unterdrückt die Expression der γ‑Globin-Kette (HBG1, HBG2) innerhalb des HBB-Genclusters. Patienten mit natürlicher Variante von BCL11A exprimieren ungewöhnlich viel HbF-Ketten. Bei transfusionsabhängiger β‑Thalassämie kann eine Hochregulierung von HbF die Notwendigkeit einer Transfusionstherapie verringern oder beseitigen, und bei der SZE könnten vasookklusive Krisen dadurch verringert werden [35]. Trotz vielversprechender Ergebnisse aus laufenden klinischen Studien fehlen für die Gentherapie noch Langzeitbeobachtungen über die Wirksamkeit und das Auftreten möglicher Nebenwirkungen.

Aufgrund von Migration aus endemischen Gebieten und aufgrund der angebotenen breiten Palette auch neuartiger Therapieoptionen werden zunehmend Patienten mit SZE in Deutschland behandelt werden. Daher wird auch deutschen Ophthalmologen das Krankheitsbild der SZR und SZM vermehrt begegnen, sodass es sich auch hierzulande lohnt, sich mit dieser interessanten Erkrankung auseinanderzusetzen.

## Fazit für die Praxis

- Die Sichelzellerkrankung (SZE) zählt zu den häufigsten Hämoglobinopathien.
- Durch zunehmende Migration aus Endemiegebieten wird die SZE in Deutschland präsenter werden und auch Ophthalmologen vermehrt begegnen.
- Retinale Komplikationen der SZE umfassen periphere nichtproliferative und proliferative Veränderungen sowie eine Makulopathie (SZM).
- Die SZM ist durch eine temporale Verdünnung der inneren Netzhautschichten gekennzeichnet. Sie findet durch Fortschritt der apparativen Diagnostik erst in den letzten 5 Jahren vermehrt Eingang in die internationale Literatur.
- Wissen um diese Form der okulären Komplikation der SZE verhindert durch eine frühzeitige Diagnosestellung unnötige Diagnostik.
